# Growth Inhibition of a Novel Iron Chelator, DpdtC, against Hepatoma Carcinoma Cell Lines Partly Attributed to Ferritinophagy-Mediated Lysosomal ROS Generation

**DOI:** 10.1155/2018/4928703

**Published:** 2018-08-05

**Authors:** Tengfei Huang, Yanjie Sun, Yongli Li, Tingting Wang, Yun Fu, Cuiping Li, Changzheng Li

**Affiliations:** ^1^Department of Molecular Biology and Biochemistry, Xinxiang Medical University, Xinxiang, Henan 453003, China; ^2^Experimental Teaching Center of Biology and Basic Medicine, Sanquan College of Xinxiang Medical University, Xinxiang, Henan 453003, China; ^3^Department of Histology and Embryology, Sanquan College of Xinxiang Medical University, Xinxiang, Henan 453003, China; ^4^Laboratory of Molecular Medicine, Xinxiang Medical University, Xinxiang, Henan 453003, China

## Abstract

Some iron chelators display significant anticancer activity that may involve ferritin degradation either in proteasomes or in lysosomes, and the latter might involve ferritinophagy with a period. However, the correlation of ferritinophagy with anticancer activity of iron chelator was not fully determined. Revealing the underlying link therefore is required. Di-2-pyridylketone dithiocarbamate (DpdtC), a novel iron chelator, could mobilize iron from ferritin and displayed excellent antitumor against hepatoma carcinoma cell lines (IC_50s_ = 0.4 ± 0.2 for HepG2 and 3.5 ± 0.3 *μ*M for Bel-7402, resp.); we speculated that the antiproliferative action of DpdtC might involve ferritinophagy. To this end, the alterations of ferritin, microtubule-associated protein light chain 3 (LC3-II), and nuclear receptor coactivator 4 (NCOA4) were investigated after exposure of DpdtC to the cells. The results revealed that DpdtC could cause increases of autophagic vacuoles and LC3-II. The data from cellular immunofluorescence and Western blotting showed a reciprocal relation between abundances of ferritin and LC3-II, but the trends of NCOA4 and LC3-II in abundance were in a similar manner, indicating that a ferritinophagy occurred. Further studies revealed that the ferritinophagy evoked an iron-driven intralysosomal oxidative reaction, resulting in LMP change and lipid peroxidation. Thus, a ferritinophagy-mediated lysosomal ROS generation playing a role in the antiproliferative action of DpdtC could be proposed, which will enrich our knowledge of iron chelator in cancer therapy.

## 1. Introduction

Iron is an essential element and plays a crucial role in cellular proliferation and DNA synthesis. In biological systems, iron acts as a cofactor in biochemical processes, such as oxygen storage, oxidative phosphorylation, and enzymatic reactions or presents as free ion (labile iron pool (LIP)) that generate reactive oxygen species (ROS) via the Fenton reaction, therefore the level of free iron in a cell must be tightly controlled [1, 2]. It has been demonstrated that the LIP is regulated by ferritin, a highly conserved iron storage protein, which is composed of two subunits, H-ferritin and L-ferritin, and the twelve pairs of subunits binding head to foot form the 24-subunit ferritin cage [3, 4]. When the iron level in the cell is low, ferritin is degraded allowing the release of iron for use by the cell.

In addition, some reductants or iron chelators could extract iron from ferritin, which was considered to be due to the small reductants or chelators entering to the interior of the ferritin molecule through three-fold channels in the protein shell, but not for larger ones [5]. Although iron chelators lead to iron poor ferritins in vitro, there is no evidence that iron can exit ferritin prior to ferritin degradation in cellular level [6]. Iron chelators can induce ferritin degradation that occurs either in lysosomes or in proteasomes. Generally, the worn-out proteins or organelle can be degraded in lysosomes, and microtubule-associated protein light chain 3 (LC3) is involved in the proteolytic process; consequently, LC3 is considered as an autophagy indicator. During autophagy, cytosolic form of LC3 (LC3-I) is conjugated to phosphatidylethanolamine to form LC3-phosphatidylethanolamine conjugate (LC3-II), which is recruited to autophagosomal membranes that engulf the damaged proteins. If nuclear receptor coactivator 4 (NCOA4) is involved in ferritin delivery to autophagosome, the proteolytic process is called ferritinophagy [7]. The dysregulation of ferritinophagy leads to change in LIP and correlates many diseases [8, 9]. Iron chelators can lead to ferritin degradation and the route of its degradation is dependent on the specificity of iron chelators [10–12]. A cancer cell has higher iron demand than normal cell; reduction of iron availability will be a favor to inhibit cancer cell proliferation. One of the mechanisms of iron chelators is a disturbance of iron homeostasis [13–17], in addition, iron chelators can also induce apoptosis, autophagy, and inactivate ribonucleotide reductase. Therefore, iron chelation has been considered as a promising strategy in cancer therapy. Although a lot of works related to iron chelators have been performed and some details in the mechanism were gained, many puzzled issues remain to be solved. Currently, only DFO can induce ferritin degradation (ferritinophagy) in lysosomes via a specific cargo, NCOA4 [7, 18], but whether other iron chelators have a similar action in inducing ferritinophagy was poorly studied. Secondly, the correlation between ferritinophagy and antiproliferative action induced by iron chelators remains to be determined. Furthermore, the relation between the redox property of an iron chelate and antiproliferative action of the ligand during ferritinophagy is poorly understood. Solving the above puzzled issues will enrich our knowledge in cancer chelation therapy. We speculate that the antiproliferative effect of iron chelator might involve ferritinophagy, and redox property of the iron chelate may be a crucial contributor in growth inhibition. In this study, we presented a study of a novel iron chelator, DpdtC (di-2-pyridylketone hydrazone dithiocarbamate), on iron mobilization and proliferation, revealing that the occurrence of ferritinophagy was one of the crucial sources for ROS generation and partly contributed to its antitumor activity.

## 2. Results

### 2.1. DpdtC Mobilized Iron from Ferritin

**Figure 1:**
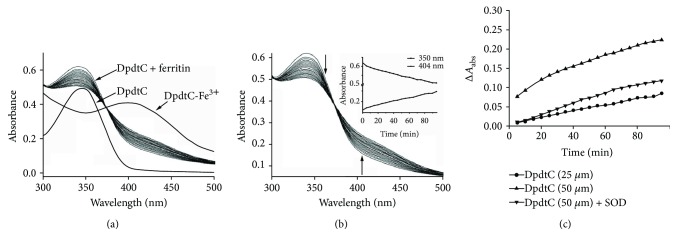
DpdtC induced iron mobilization from ferritin. (a) The absorbance spectrum of DpdtC and its iron complex; the profiles of iron extracting from ferritin by DpdtC were also included; (b) the spectral changes of ferritin during addition of DpdtC. The insert showed the absorbance changes at specific wavelengths (350 and 404 nm); (c) iron mobilization from ferritin by different concentrations of DpdtC was assayed by measuring the absorbance difference at 404 nm, and effect of SOD on iron mobilization was conducted in a similar way.

Previously, we reported DpdtC having the ability in catalase inhibition that may partly contribute to the ROS production [19], which hinted that a Fenton reaction might occur, contributing its antiproliferative action. To extend our knowledge related to the compound (see Supplementary Materials, Figure S1a), in the present study, we assessed the metal chelating ability of DpdtC with iron. And the stoichiometric ratio between DpdtC and ferric chloride was determined by Job's method of continuous variations (see Supplementary Materials). As shown in Figure S1, a new peak around 404 nm was indicative of complex formation of the DpdtC with iron (III) (Figure S1c). Accordingly, the composition of the iron complex was also determined (Figure S1d). Similarly, DpdtC could also chelate ferric iron in a similar manner (see Supplementary Materials, Figure S1b). Next, we tested whether DpdtC as like other tridentate chelators could mobilize iron from ferritin; to this end, a purified ferritin was normally used in the assay. Because micromolar concentration required in DpdtC induced growth inhibition (see Section 2.2), thus a low concentration was chosen in the assay of iron mobilization (direct extraction by chelators generally required much higher concentration (3.5–100 mM) to diffuse the Fe(III)-chelate out of the ferritin shell) [20]. To monitor the iron release from ferritin, the MLCT (metal-to-ligand charge-transfer) absorption (at ~404 nm) that corresponded to Fe(III)-DpdtC complexes was measured during the time course (Figure 1(a)). As shown in Figure 1(a), the absorbance intensity of ferritin at 350 nm was decreased, but the absorbance at 404 was increased with increasing incubation time, their changes are shown in the insert (Figure 1(b)). To determine whether superoxide radical was involved in the iron mobilization, the superoxide dismutase (SOD) was added, showing that SOD inhibited significantly the iron release, thus indicating the involvement of reactive oxygen species (ROS) in the process of iron removal from ferritin (Figure 1(c)), implying an oxygen-catalytic iron mobilization might be involved, which was similar to that previously reported [20, 21].

### 2.2. The Antiproliferative Activity of the DpdtC

**Figure 2:**
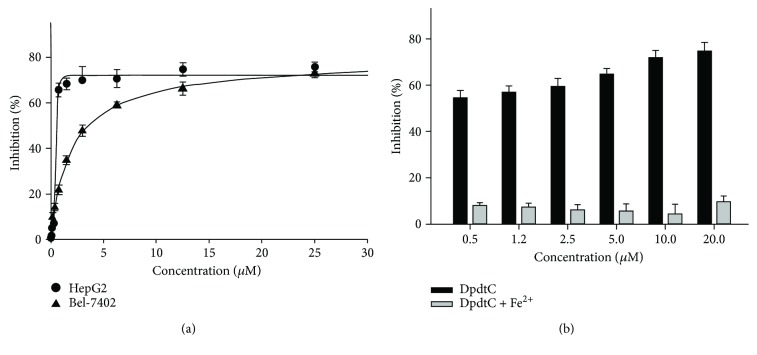
Proliferation inhibition of DpdtC and its iron complex against hepatoma carcinoma cell lines: (a) proliferation inhibition of DpdtC against HepG2 and Bel-7402 cell lines; (b) antiproliferative action of DpdtC against HepG2 cell was attenuated by the addition of iron.

It has been reported that some iron chelators displayed significant proliferation inhibition against tumor cell lines, thus the effect of DpdtC on the proliferation of HepG2 and Bel-7402 cell lines was evaluated based on MTT method. The dose-response curves are depicted in Figure 2. As expected, DpdtC had a significant growth inhibition for HepG2 and Bel-7402 cells (IC_50_: 0.4 ± 0.2 for HepG2 and 3.5 ± 0.3 *μ*M for Bel-7402). To determine the cytotoxicity of DpdtC to a normal cell, the normal human hepatic cell line LO2 was used for comparison.  Figure S2 showed that DpdtC also exhibited a significant growth inhibition against the normal human hepatic cell, with IC_50_ = 3.15 ± 0.24 *μ*M, indicating that the selectivity of the agent was not obvious even greater than that of HepG2 cell in IC_50_ value. Since that DpdtC was able to chelate iron, the effect of iron ion on the proliferative action was also determined; unexpectedly, the iron (II) addition (equal mole) significantly attenuated the antiproliferative activity of DpdtC against HepG2 cell (Figure 2(b)), but the addition of copper ion significantly enhanced its activity (data not shown), indicating that iron (copper) ions could affect the manner of action of DpdtC due to its chelating ability.

### 2.3. DpdtC Induced ROS Generation

**Figure 3:**
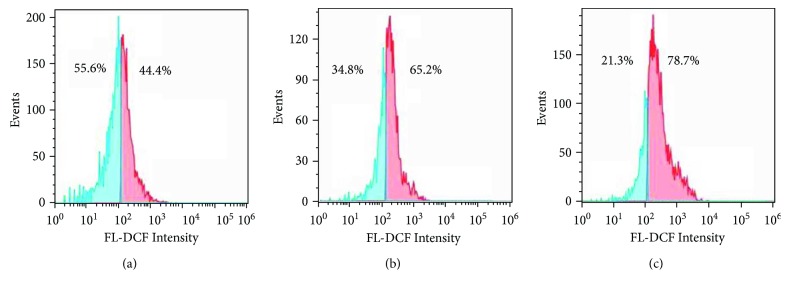
DpdtC induced ROS production in HepG2 cell. (a) Control; (b) 1 *μ*M DpdtC; (c) 2 *μ*M DpdtC.

It has been well documented that most therapeutic drugs in the mechanism can induce production of reactive oxygen species (ROS). The excellent antiproliferative action of DpdtC spurred us to investigate the underlying mechanism. Thus, the cellular ROS level was measured by flow cytometry after the cells were stained by a ROS dye, dichlorofluorescein (DCF). In view of higher inhibitory effect on HepG2 cell, the assay was only conducted on this cell line. As shown in Figure 3, the populations in higher fluorescence intensities significantly increased by 21~34% after exposure of DpdtC to the cells for 12 h (a fluctuated pattern of ROS was observed after 48 h incubation, Figure S3), hinting that ROS production was involved in the antiproliferative action.

### 2.4. DpdtC Exposure Resulted in the Accumulation of Autophagic Vacuole

**Figure 4:**
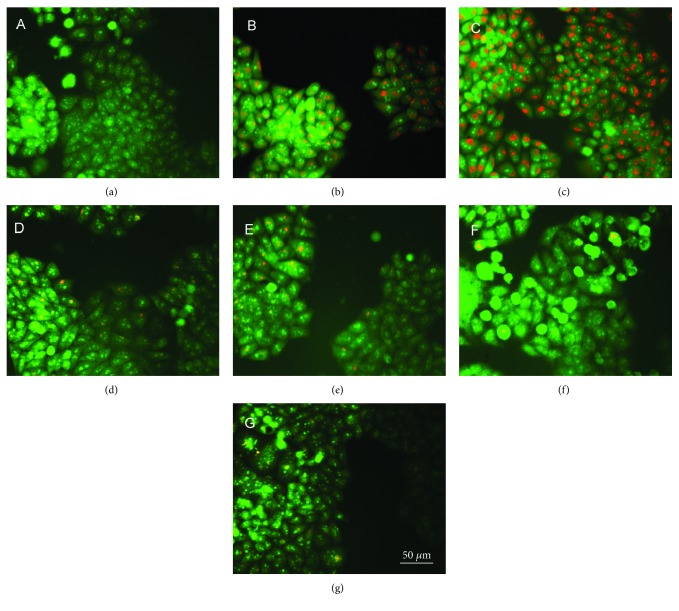
DpdtC induced formation of acidic vesicular organelles autophagy; the HepG2 cells were treated by the indicated agents for 24 h. (a) Control; (b) 1 *μ*M DpdtC; (c) 2 *μ*M DpdtC; (d) 1 *μ*M DpdtC + 3-MA; (e) 2 *μ*M DpdtC + 3-MA(2.5 mM); (f) 1 *μ*M DpdtC + Fe^2+^(1 *μ*M); (g) 2 *μ*M DpdtC + Fe^2+^(2 *μ*M), scale bar: 50 *μ*m.

Normally, ROS production associated autophagy. Based on ROS assay from HepG2 cell, we speculated that the autophagy might involve DpdtC induced growth inhibition. The formation of acidic vesicular organelles (AVOs) is a characteristic marker in the process of autophagy [22, 23]. Thus, the AVOs were assayed by acridine orange staining.  Figure 4 clearly showed that the fluorescence intensity of granular acridine orange (red in acidic vesicular organelles) increased (Figures 4(b) and 4(c)), implying autophagy occurrence. The addition of an autophagy inhibitor, 3-methyladenine (3-MA), could significantly attenuate the fluorescence intensities of AVOs, supporting above deduction. Interestingly, the addition of iron ((NH_4_)_2_Fe(SO_4_)_2_) also attenuated the fluorescence intensities of AVOs, indicating that iron as like 3-MA led to decrease in AVOs formation. In addition, iron also attenuated the antiproliferative action of DpdtC (Figure 2(b)). Those implied the alteration of DpdtC action upon addition of iron, and it was related to autophagy (Figures 4(f) and 4(g)).

**Figure 5:**
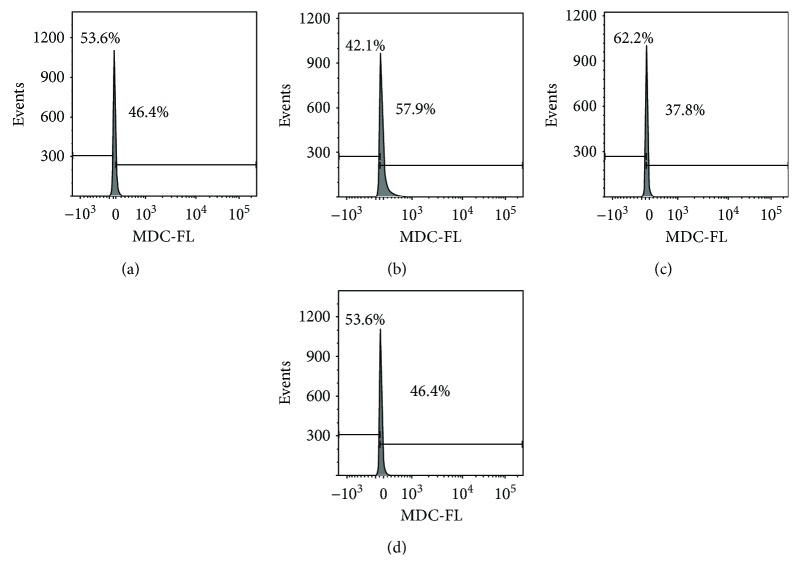
MDC staining for determination of autophagosomes induced by DpdtC. A similar protocol was used in the determination of AVOs via acridine orange staining, but in this assay, MDC and flow cytometry were used. (a) Control; (b) 2 μM DpdtC; (c) 2 *μ*M DpdtC + 3-MA(1.5 mM); (d) 2 *μ*M DpdtC + NAC (1.5 mM).

The alteration in AVOs or autophagosomes was further determined by monodansylcadaverine (MDC) staining via flow cytometry technique [24]. As shown in Figure 5, DpdtC exposure led to 11.5% increase in higher fluorescence population compared to without treatment of the agent, but the fluorescence intensities were significantly attenuated by the addition of 3-MA (Figures 5(a) and 5(c)), supporting that the accumulation of autophagic vacuole was stemmed from DpdtC stimulus. Similar results were observed in the microscopic analysis (Figure S4). Furthermore, NAC could neutralize the effect of DpdtC (Figure 5(d)), implying that ROS played an important role in autophagy induction.

### 2.5. DpdtC Exposure Induced Ferritinophagy

**Figure 6:**
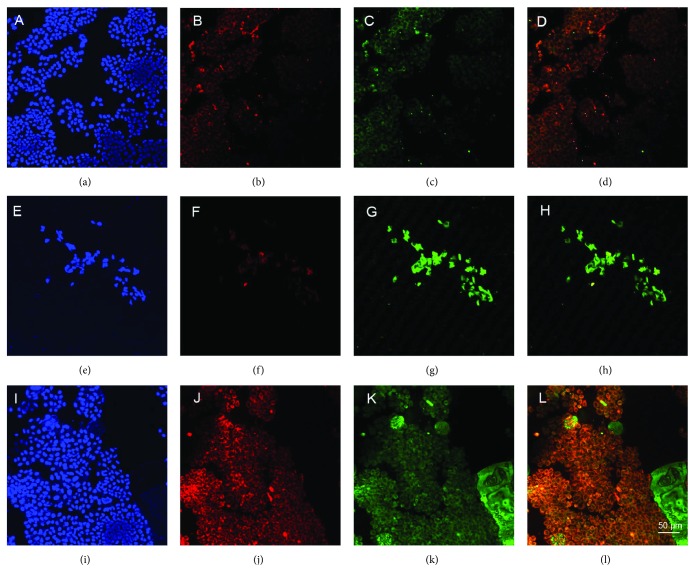
DpdtC induced ferritin autophagy (ferritinophagy). The nuclei stained by DAPI in blue, ferritin labeled in red, and LC3 labeled in green. (a–d) Control group; (a) nuclei in blue; (b) ferritin in red; (c) LC3 in green; (d) merging of ferritin with LC3. (e–h) DpdtC treated group: (e) nuclei in blue; (f) ferritin in red; (g) LC3 in green; (h) merging of ferritin with LC3. (i–l) DpdtC combined with 3-MA group: (i) nuclei in blue; (j) ferritin in red; (k) LC3 in green; (l) merging of ferritin with LC3, scale bar: 50 *μ*m.

The cellular ROS production is mainly from the mitochondrial, while the labile iron pool (LIP) was able to promote the formation of reactive oxygen species (ROS). And LIP and ROS levels were shown to follow similar “rise and fall” patterns [25]. Next, we questioned that the rise in ROS may also indicate higher LIP content after DpdtC exposure to the HepG2 cell and correlate ferritin (FT) degradation. In view of the fact that the FT degradation may occur via autophagy, the changes of cellular ferritin and autophagic marker, LC3 was monitored via immunofluorescence technique. As shown in Figure 6, the upregulated LC3 and downregulated ferritin were observed (Figures 6(a) and 6(f)), indicating that the ferritin degradation was via autophagic proteolysis. To further confirm the above conclusion, the 3-MA, an inhibitor in the formation of autophagic vacuole was added during exposure of DpdtC to the HepG2 cells. As expected, the ferritin degradation was significantly attenuated (Figures 6(g) and 6(i)), confirming that the downregulated ferritin was stemmed from autophagic degradation.

**Figure 7:**
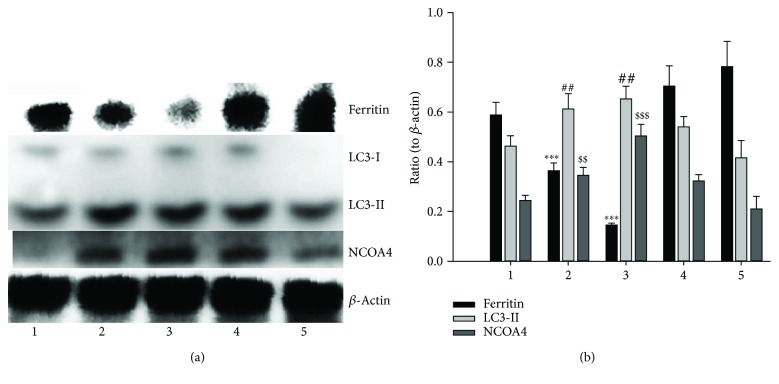
DpdtC induced ferritinophagy. (a) Western blot analysis; (b) quantification analysis. The data were obtained from three independent Western blots based on comparison of the investigated protein with *β*-actin. (1) Control (water); (2) 1 *μ*M DpdtC; (3) 2 *μ*M DpdtC; (4) 1 *μ*M DpdtC + 3-MA (1.5 mM); (5) 2 *μ*M DpdtC + 3-MA (1.5 mM). (^∗∗∗,$$$^ *p* < 0.01; ^##,$$^ *p* < 0.05; one-way ANOVA).

Since the ferritin degradation was via autophagy, the ferritinophagy might occur. To test the hypothesis, the specific carrier for ferritinophagy, NCOA4, and ferritin as well as LC3 were determined by Western blotting. As shown in Figure 7, the ferritin and LC3-I were decreased with increased DpdtC, but the LC3-II and NCOA4 were increased with increased DpdtC, clearly indicating that DpdtC could induce ferritinophagy, which was similar to DFO in ferritinophagy induction [7].

### 2.6. The DpdtC Induced Change in Lysosomal Membrane Permeability (LMP)

**Figure 8:**
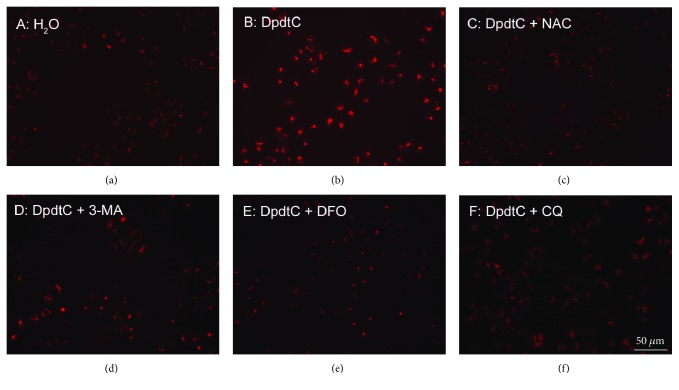
DpdtC induced change in lysosomal membrane permeability (LMP). LysoTracker Red stained HepG2 cells: (a) control; (b) 1.0 *μ*M DpdtC; (c) 1.0 *μ*M DpdtC + NAC (1.5 mM). (d) 1.0 *μ*M DpdtC + 3-MA (1.5 mM); (e) 1.0 *μ*M DpdtC + DFO (50 *μ*M); (f) 1.0 *μ*M DpdtC + CQ (50 *μ*M). Scale bar: 50 *μ*m.

Since DpdtC induced ferritinophagy that occurred in lysosomes, the iron release from digested ferritin would synchronously occur. Next, the ferric would be reduced by the reducing agents, which triggered Fenton reaction, producing massive ROS that caused an alteration in LMP. To test the hypothesis, LysoTracker Red that can accumulate within lysosomes was employed to assess the lysosome membrane permeability [13]. As shown in Figure 8, the red fluorescence intensities of HepG2 cells were increased with treatment of DpdtC compared to nontreatment of the cells, indicating that more LysoTracker Red accumulated in lysosomes and LMP was altered (Figure 8(b)). However, the addition of NAC could attenuate the alteration of LMP, hinting that the change correlated to ROS production. Similar situations were observed when addition of 3-MA or chloroquine (Figures 8(d) and 8(f)), indicating that autophagy inhibition could attenuate the alteration in LMP. Interestingly, DFO could also significantly attenuate fluorescence intensities of LysoTracker Red in lysosomes, demonstrating that the Fenton reaction derived from ferritinophagy could be quenched by DFO (Figure 8(e)) due to redox inactive DFO-iron chelate. This clearly indicated that lysosomal ROS were stemmed from iron release from ferritinophagy. The quantification analysis of fluorescence changes in different groups was shown in Figure S5.

### 2.7. Upregulation of TRPML1 Correlated to Autophagy

**Figure 9:**
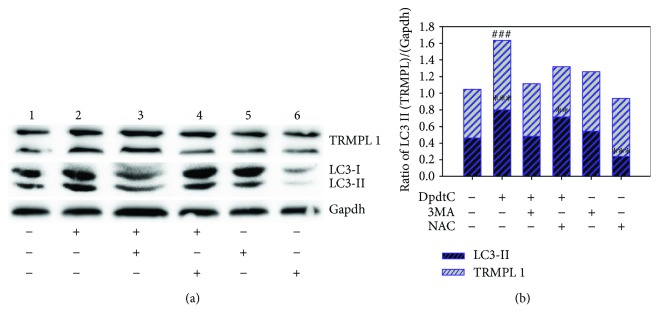
The effect of lysosomal ROS on autophagy and TRMPL1. A: Western blot analysis; B: quantification analysis. The data were obtained from three independent Western blots based on comparison of the investigated protein with Gapdh (1) control; (2) 1 *μ*M DpdtC; (3) 1 *μ*M DpdtC + 1.5 mM 3-MA; (4) 1 *μ*M DpdtC + 1.5 mM NAC; (5) 1.5 mM 3-MA; (6) 1.5 mM NAC. (^∗∗∗,###^ *p* < 0.01; ^∗∗^ *p* < 0.05; one-way ANOVA).

TRPML1 (transient receptor potential cation channel, mucolipin subfamily, member 1) is a cation-permeable channel that is predominantly localized on the membranes of late endosomes and lysosomes [26]. TRPML1 may mediate both the release of Ca^2+^ and heavy metal Fe^2+/^Zn^2+^ ions into the cytosol from the lysosomes [27]; thus, TRPML1 was considered as a lysosomal ROS sensor [28]. Next, we assayed TRPML1 expression when DpdtC was exposed to the HepG2 cells. As expected, DpdtC led to upregulation of TRPML1 (Figure 9(a)), but downregulation of TRPML1 was observed by addition of 3-MA or NAC (Figure 9(a)), indicating that the lysosomal ROS was stemmed from the occurrence of ferritinophagy, in consistent with the changes in LC3-II. The quantitative analyses of TRPML1 and LC3-II are shown in Figure 9(b).

### 2.8. Lipid Peroxidation Occurred When DpdtC Was Exposed to HepG2 Cell

**Figure 10:**
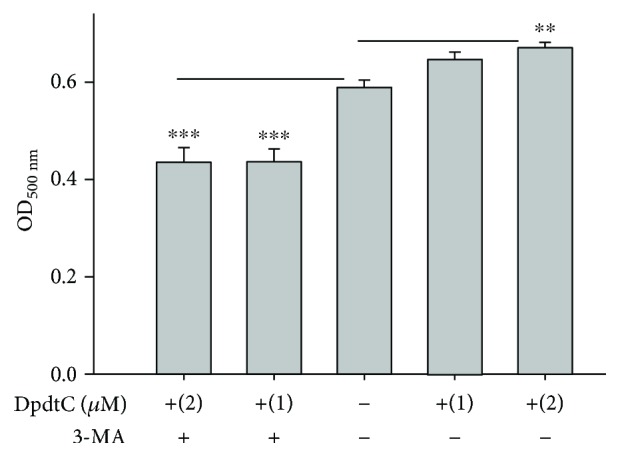
A lysosomal ROS caused lipid peroxidation. The absorbance at 500 nm represents the peroxidative degree of lipid; clearly, DpdtC induced lipid peroxidation, but 3-MA could attenuate the oxidative effect, indicating that the lipid peroxidation was related to autophagy. (^∗∗∗^ *p* < 0.01; ^∗∗^ *p* < 0.05, one-way ANOVA).

As mentioned above, the cellular ROS were increased when DpdtC was exposed to the HepG2 cells. The intracellular ROS at least partly originated from lysosome due to the occurrence of ferritinophagy that may involve Fenton-like reactions [29]. Excess ROS lead to damage biological macromolecules, including lipid peroxidation. To test this hypothesis, a lipid peroxidation assay was performed as described previously [30]. As shown in Figure 10, DpdtC significantly enhanced lipid peroxidation in a concentration-dependent manner, but the addition of 3-MA drastically attenuated the lipid peroxidation, consistent with the results from ferritinophagy.

## 3. Discussion

A widely accepted concept is that iron chelators can coordinate iron from cytosolic labile iron pool (LIP), leading to iron depletion that provokes the association of iron response proteins with iron response element (IRE) in UTRs (untranslated regions) of various mRNAs whose products are involved in iron metabolism [31]. Iron chelators are also capable to mobilize iron from ferritin in vitro, leading to form an iron poor ferritin, but the evidence of iron mobilization by chelator in cellular level is still lacking. A recent study showed that ferritin-Fe mobilization does not occur through changes in cellular concentrations of reducing/chelating agents but by the coordinated movement of ferritin and DMT1 to lysosomes [32], which causes ferritin degradation in the lysosome. Iron chelators can sequester iron, but the redox potentials of the resulting complexes (iron) varied with association constant. The iron chelate can serve as either a reductant or an oxidant, depending on the redox potential of its opponent encountered; therefore, iron chelator has two sides, either as an antioxidant or as a prooxidant. However, *in vivo* (or cellular level) this situation is more complicated due to different cellular locations of the iron chelates. Generally, iron chelators function as either an antioxidant or a prooxidant, depending on the redox nature of iron complex formed [33, 34]; however, the direct correlation between ROS production and viability remains to be determined. Many iron chelators, such as deferoxamine (DFO), deferiprone, and deferasirox, caused ferritin degradation, but only DFO-induced ferritin loss was prevented by chloroquine treatment, indicating that DFO-induced proteolysis occurred in lysosomes; the others led to ferritin degradation in proteomes, which depended on the specificity of the chelators [10, 11]. In addition, iron chelators also displayed excellent antitumor activities [35]; the representative chelators are heterocyclic carboxaldehyde thiosemicarbazones (Dp44mT), analogs of pyridoxal isonicotinoyl hydrazine (PIH), tachpyridine, o-trensox, and other natural products (DFO, desferrithiocin), and many of them are at various stages of clinical trials [36, 37]. However, some disadvantages of iron chelators such as shorter plasma half-life (DFO) and high toxicity in the kidney and neurological problems have motivated the scientific community to find new iron ligands [35, 38]. In the present study, we presented the investigation of a novel dithiocarbamate derivative, DpdtC, on the characteristic of iron mobilization and antiproliferative activity against hepatoma carcinoma cell lines (Figures 1 and 2). The excellent antiproliferative action of DpdtC promoted us to determine the underlying mechanism (Figure 2). Data revealed that DpdtC, as other iron chelators, could induce ROS production (Figure 3) [35], triggering either physiologic or cytotoxic autophagy. Due to multiple steps in autophagy occurrence, an important step is autophagosome formation; thus, the alteration in autophagic vacuoles when DpdtC was exposed to HepG2 cell by both acridine orange and MDC staining was investigated. As expected, the autophagic vacuoles increased after exposure of DpdtC to HepG2 cell but significantly decreased when combined with either NAC or 3-MA (Figures 4 and 5 and Figure S3), indicating that the manner of action of DpdtC involved autophagy and ROS production. Many chelators can induce ferritin degradation that may occur in lysosomes, thus the level of cytoplasmic ferritin was determined. As expected, a decrease in ferritin and an increase in LC3-II were observed (Figure 7), implying that the ferritin degradation occurred in lysosomes. Since ferritin degradation was associated with autophagy, this process might be also involved in ferritinophagy. To support this hypothesis, the status of specific cargo for ferritinophagy, NCOA4, required to be determined (Figure 7). As expected, accompanied by downregulated ferritin an upregulated NCOA4 was founded, indicating that ferritinophagy indeed occurred. This is the first report that DpdtC, a dithiocarbamate derivative, induced ferritinophagy except DFO [7]. Owing to the occurrence of ferritinophagy, the abundance of lysosomal iron increased; consequently, an increase in LIP, oxidative stress, and cell death may come up [39]. The consequence of ferritinophagy inevitably led to lysosomal destruction. Figure 8 showed that more LysoTracker Red dyes were accumulated in lysosomes after exposure of DpdtC to HepG2 cells, but this accumulation could be attenuated by the addition of NAC (Figure 8(c)), 3-MA, or chloroquine, indicating that the damage of lysosomal membrane (or integrity) was caused by ROS and ferritinophagy [21, 40]. It was imagined that the ferric iron was liberated from digested ferritin and reduced further by the endosomal ferrireductase Steap3 in the acidified lysosome [41]; the resulting ferrous ion triggered Fenton reaction. To further confirm that the lysosomal ROS were stemmed from Fenton reaction, DFO, an iron chelator (lysophilic chelator) that can protect the lysosome membrane from oxidative damage was employed [42]. As shown in Figure 8(e), DFO efficiently decreased the accumulation of LysoTracker Red dyes, quenching Fenton reaction. During ferritinophagy, iron leaking from the lysosomes to the cytosol may also occur either via DMT-1 or lysosomal iron channel TRPML1 that was considered as a lysosomal ROS sensor [43, 44]; therefore, the expression of TRPML1 reflected the status of lysosomal ROS and iron leaking. As expected (Figure 9), an upregulated TRPML1 was observed, but the addition of NAC could downregulate TRPML1, indicating that the cell death induced by DpdtC was a lysosomal ROS dependent. Excess ROS leading to lipid peroxidation, proteins, and DNA damage has been well documented [45]; the lipid peroxidation correlated with excess ROS production. Figure 10 showed that DpdtC induced an increase in lipid peroxidation, but the peroxidation could be attenuated by the addition of 3-MA, implying that autophagy was a crucial contributor in growth inhibition [46]. It should be noted that the autophagy (ferritinophagy) and growth inhibition induced by DpdtC could be eliminated by addition of iron ion (Figures 2 and 4); a reasonable explanation for this may be due to ability loss in moving ferritin to lysosome upon DpdtC chelates iron [32], and a much higher cytoplasmic DpdtC concentration than iron in LIP may ensure its ability in ferritinophagy induction.

Taken together, the growth inhibition against hepatic cancer induced by DpdtC was partly correlated with ferritinophagy that triggered lysosomal ROS production via Fenton reaction and iron release from lysosomes to cytoplasm. Obviously, the occurrence of ferritinophagy was prerequisite; secondly, the redox activity of iron chelate (redox property) and location of chelator in the cell may be crucial factors, which determined subsequent biological effects, such as growth inhibition. However, insight into the correlation between redox nature of iron chelate and induced growth inhibition requires more studies in the future due to the diversity of iron chelators in structure and complex interactions with biological molecules.

## 4. Materials and Methods

### 4.1. Materials

All reactants and solvents were analytical reagents (AR) grade. 3-(4,5-dimethylthiazol-2-yl)-2,5-diphenyltetrazolium bromide (MTT), monodansylcadaverine (MDC), 3-methyladenine (3-MA), chloroquine, dichlorofluorescein (H_2_DCF-DA), deferoxamine (DFO), 4′,6-diamidino-2-phenylindole (DAPI), Roswell Park Memorial Institute (RPMI) 1640, Horse spleen ferritin, and other chemicals were purchased from Sigma-Aldrich. LC3 and ferritin antibody were obtained from Proteintech Group (Wuhan, China). Nuclear receptor coactivator 4 (NCOA4) antibody was purchased from Boster Biological Technology Co. Ltd. (Wuhan, China), and superoxide dismutase (SOD) was obtained from Beyotime Biotechnology (Beijing, China). Transient receptor potential cation channel, mucolipin subfamily, member 1 (TRPML1) antibody was purchased from Abcam (Shanghai, China). Secondary antibodies (or fluorescence labeled) were obtained from EarthOx, LLC (San Francisco, USA).

### 4.2. Iron Mobilization by DpdtC

The iron release experiments were conducted in 0.05 M Tris-HCl, 50 mM NaCl, pH 7.4, 10 *μ*l ferritin (12.5 mg/ml), and varied concentration of DpdtC (or with 3 unit SOD) in a total of 1 ml volume. The kinetics of iron release from ferritin was monitored by the increase in the characteristic of the MLCT (metal-to-ligand charge-transfer) absorption bands of the corresponding iron (II)-chelate complexes (404 nm for DpdtC). The absorbance was measured every 5 min on a Shimadzu UV-2450 spectrophotometer with thermostatic circulating device at 37°C.

### 4.3. Cytotoxicity Assay (MTT Assay)

The stock solution of DpdtC (10 mM) was prepared in autoclaved deionized water and diluted to the required concentration with culture when used. HepG2 cells (or Bel-7402, LO2) were cultured in RPMI 1640 medium supplemented with 10% fetal calf serum (FCS) and antibiotics. The cells in exponential phase were collected and seeded equivalently into a 96-well plate; next, the varied DpdtC (or in the presence of equivalent iron (II) salt) was added after the cells adhered. Following 48 h incubation at 37°C in a humidified atmosphere of 5% CO_2_, 10 *μ*l MTT solution (5 mg/ml) was added and further incubated for 4 h. The cell culture was removed by aspiration, and 100 *μ*l DMSO was added in each well to dissolve the formazan crystals. The measurement of absorbance of the solution that was related to the number of live cells was performed on a microplate reader (MK3, Thermo Scientific) at 570 nm. Percent growth inhibition was defined as percent absorbance inhibition within appropriate absorbance in each cell line. The same assay was performed in triplet.

### 4.4. Flow Cytometry Analysis of Cellular ROS

Similar to MTT assay, the HepG2 cells were treated by either DpdtC or in combined with 3-MA (or NAC) for 24 h. The cells were collected by centrifugation after trypsinization. Following by PBS washing, the cells pellet was resuspended in H_2_DCF-DA containing serum-free culture medium and incubated for 30 min. Next, after removing the H_2_DCF-DA contained medium by centrifugation and washing with PBS, the cells were finally resuspended in PBS. The intracellular ROS assay was performed on a flow cytometer (Becton-Dickinson, USA).

### 4.5. Flow Cytometric and Microscopic Analyses of Autophagic Vacuoles

Cells were seeded into a 6-well plate and treated as described above for the cell viability assay. The cells were treated with either the agent alone (1.0 or 2.0 *μ*M) or a combination with 3-MA (1.5 mM) or NAC (1.5 mM)) for 24 h. Then, the cell culture was removed, following PBS washing, trypsin digestion; finally, the MDC (50 μM) were added as described previously [47]. The stained cells were subjected to flow cytometric analysis (Becton-Dickinson, USA).

### 4.6. Immunofluorescence Analysis

HepG2 cells were first cultured in a 6-well plate with a cover glass overnight. Following DpdtC treatment for 24 h, the cells were first fixed with 4% paraformaldehyde in PBS for 15 min at 37°C, and then permeabilized with 0.2% Triton X-100 in PBS for 10 min. After blocking with 1% BSA in PBS for 30 min, the cells were incubated with LC3 and ferritin (H chain) primary antibody based on dilution recommended by the company; at 4°C the plate was shaken overnight. Next, removing the primary antibodies and washing with PBS, the cells were further incubated with fluorescence-labeled secondary antibody for 3 h at room temperature. After removing the secondary antibody, the cells were further counterstained with DAPI. Finally, a confocal laser scanning microscope (LSM 410, Zeiss, Jena, Germany) was used to visualize the cells; the representative cells were selected and photographed.

### 4.7. Autophagy and Lysosomal Membrane Permeability (LMP) Affected by DpdtC

The alteration of LMP was assayed as previously described [13]. For the detection of the acidic cellular compartment, acridine orange (or LysoTracker Red; Invitrogen) was used, which emits bright red fluorescence in acidic vesicles but green fluorescence in the cytoplasm and nucleus. After treatment of the cells with the agent, acridine orange was then added at a final concentration of 1 *μ*g/ml (the concentration of LysoTracker Red, as recommended) for a period of 15 min. Following PBS washing, the fluorescent micrographs were captured using an inverted fluorescence microscope.

### 4.8. Western Blotting Analysis

Briefly, 1 × 10^7^ HepG2 cells, treated with or without DpdtC, were scraped in lysis buffer (50 mM Tris-HCl, pH 8.0, 150 mM NaCl, 1.0% NP-40, 10% glycerol, and protease inhibitors) and subjected to sonication, followed by centrifugation at 14,000 × g. The clear supernatant was stored at −80°C. Protein concentration was determined using a colorimetric Bio-Rad DC protein assay using the MK3 microplate reader at 570 nm. Proteins (30 *μ*g) were separated on a 13~15% sodium dodecyl sulfate-polyacrylamide gel at 200 V for 3 h. The separated proteins were subsequently transferred onto a PVDF membrane at 60 V for 2 h. The membrane was washed three times with Tris-buffered saline (TBS) and then blocked for 2 h in TBS containing 0.1% Tween-20 and 5% nonfat skimmed milk. The membrane was incubated at 4°C overnight with the appropriate primary antibody used at a dilution of 1 : 300 in TBS plus 0.1% Tween-20 (TBST). The membrane was then washed several times with TBST and subsequently incubated with the appropriate HRP-conjugated secondary antibody (1 : 2000 in TBST) for 1 h at room temperature. Following washing with TBST, the protein bands were detected using a super sensitive ECL solution (Boster Biological Technology Co. Ltd.) and visualized using a Syngene G:BOX imager (Cambridge, United Kingdom). Quantifications of protein bands intensities and fluorescence intensity were performed using ImageJ software.

### 4.9. Lipid Peroxidation Assay

Lipid peroxidation analysis was performed based on spectrophotometry, in which the ferrous ion is oxidized by lipid hydroperoxides to the ferric ion and subsequently reacts with thiocyanate to form a colored complex [30]. The details were as previously described [48]. Briefly, the trypsinized cells were collected and treated with the DpdtC for 12 h; the supernatant was removed by centrifugation and washed with PBS. The peroxidized lipid was extracted using deoxygenated CHCl_3_/MeOH (2 : 1, *v*/*v* mixture, 1000 *μ*l), and the lipids were transferred to a 5 ml volumetric flask, which contained 100 *μ*l of ferrous sulfate (0.2 M HCl) and 100 *μ*l of 3% deoxygenated thiocyanate (methanol) for 60 min. Finally, deoxygenated CHCl_3_/MeOH solvents were added to the given volume. The absorbances at 500 nm were measured using a UV-2450 spectrophotometer (Shimadzu Co. Ltd., Suzhou, China). The molar absorptivity of the ferric thiocyanate complex expressed per mol of LOOH was determined to be 58,440 M^−1^ cm^−1^ [30].

### 4.10. Statistical Analysis

Data were analyzed with Prism 5.0 (GraphPad Software Inc., USA). Comparisons were made using a one-way analysis of variance or a two-tailed t-test. Results are presented as the mean ± SEM. A *p* value < 0.05 was considered statistically significant.

## Data Availability

The data used to support the finding of this study are included in the article.

## Supplementary Materials

Supplementary Materials
*Determination of the molar ratio of DpdtC to iron complex*: The reaction stoichiometry between DpdtC and (NH_4_)_2_Fe(SO_4_)_2_ was studied by Job's method of continuous variations (13). For this method, different volumes (0, 20, 40, 60, 80, 100, 120, 140, 160, and 180 *μ*l) of 1 mM (NH_4_)_2_Fe(SO_4_)_2_ were added with different volumes (200, 180, 160, 140, 120, 100, 80, 60, 40, and 20 *μ*l) of 1 mM DpdtC and diluted with water in 5 ml standard volumetric flask. The absorbance was recorded on a Shimadzu UV-2450 spectrophotometer. The absorbance at 404 nm against the mole fraction of the mole ratio of iron (II) (or DpdtC) was plotted and regressed linearly (SigmaPlot 10.0). As described in experimental section of determination of the molar ratio of DpdtC to Fe(II) complex, the molar ratio of DpdtC to Fe(III) was determined by similar method. In this protocol, the ferric chloride was used, and the titration was conducted in acetonitrile (Figure S1). *Microscopic analyses of autophagic vacuoles*: Cells were seeded into a 6-well plate and treated as described above for the cell viability assay. For morphologic analysis, autophagic vacuoles were labeled with MDC by incubating the cells that treated by either the agent or combination with 3-MA (or NAC) with 50 *μ*M MDC in PBS at 37°C for 10 minutes. After incubation, the cells were washed four times with PBS and immediately analyzed by fluorescence microscopy using an inverted microscope (Shanghai Lengguang Technology Co., Ltd., Shanghai, China) (Figure S3).
