# Supplementary material for: Growth Inhibition of a Novel Iron Chelator, DpdtC, against Hepatoma Carcinoma Cell Lines Partly Attributed to Ferritinophagy-Mediated Lysosomal ROS Generation
Source: Oxid Med Cell Longev. 2018 Aug 5;2018:4928703. doi: 10.1155/2018/4928703 (PMC6098875; doi:10.1155/2018/4928703)
Supplement: Supplementary Materials — Determination of the molar ratio of DpdtC to iron complex: The reaction stoichiometry between DpdtC and (NH4)2Fe(SO4)2 was studied by Job's method of continuous variations (13). For this method, different volumes (0, 20, 40, 60, 80, 100, 120, 140, 160, and 180 μl) of 1 mM (NH4)2Fe(SO4)2 were added with different volumes (200, 180, 160, 140, 120, 100, 80, 60, 40, and 20 μl) of 1 mM DpdtC and diluted with water in 5 ml standard volumetric flask. The absorbance was recorded on a Shimadzu UV-2450 spectrophotometer. The absorbance at 404 nm against the mole fraction of the mole ratio of iron (II) (or DpdtC) was plotted and regressed linearly (SigmaPlot 10.0). As described in experimental section of determination of the molar ratio of DpdtC to Fe(II) complex, the molar ratio of DpdtC to Fe(III) was determined by similar method. In this protocol, the ferric chloride was used, and the titration was conducted in acetonitrile (Figure S1). Microscopic analyses of autophagic vacuoles: Cells were seeded into a 6-well plate and treated as described above for the cell viability assay. For morphologic analysis, autophagic vacuoles were labeled with MDC by incubating the cells that treated by either the agent or combination with 3-MA (or NAC) with 50 μM MDC in PBS at 37°C for 10 minutes. After incubation, the cells were washed four times with PBS and immediately analyzed by fluorescence microscopy using an inverted microscope (Shanghai Lengguang Technology Co., Ltd., Shanghai, China) (Figure S3). [file 4928703.f1.pdf]

Supplementary materials

DpdtC chelates iron and forms a complex at 1:1 molar ratio

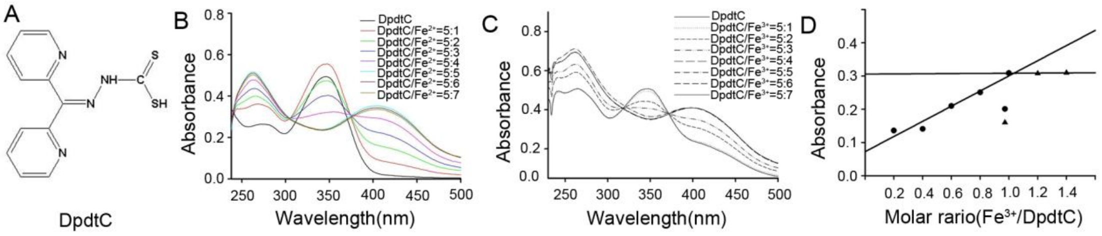

Fig. S1 UV-visible spectra of DpdtC iron complex and relation between absorbance and molar ratio. (A) structure of DpdtC; (B) Spectra of DpdtC and in the presence of varied concentration of Fe^{2+}; (C) Spectra of DpdtC and in the presence of varied concentration of Fe^{3+}; the molar ratio as indicated in the figure. (D) Plot of the absorbance of copper complex at 404 nm vs. molar ratio of Fe^{3+}/DpdtC.

DpdtC induced growth inhibition against normal human hepatic cell

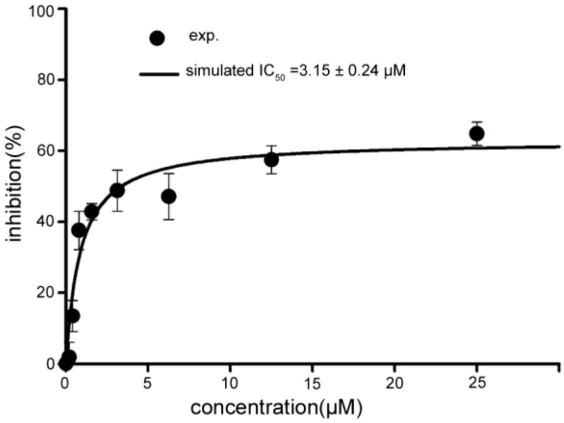

Fig. S2 DpdtC induced growth inhibition against normal human hepatic cell LO2.

ROS generation during the time period of exposure of DpdtC to HepG2 cell

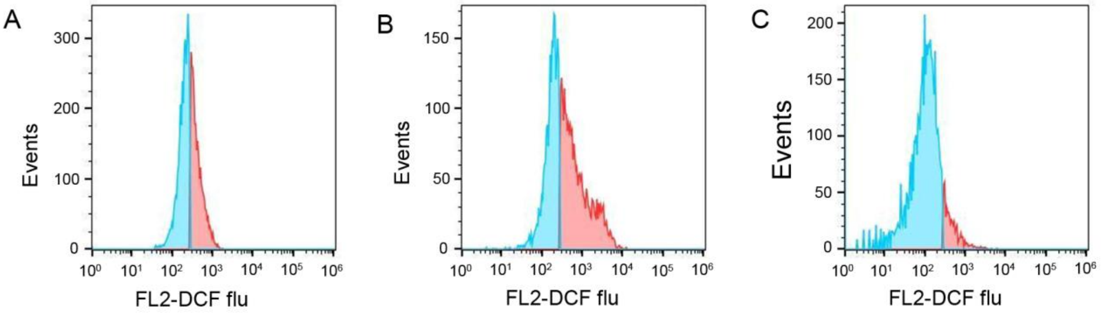

Fig. S3 DpdtC induced ROS after 48 h incubation. (A) H_2O; (B) 0.75 μM DpdtC; (C) 1.5 μM DpdtC.

DpdtC induced change in autophagic vacuoles

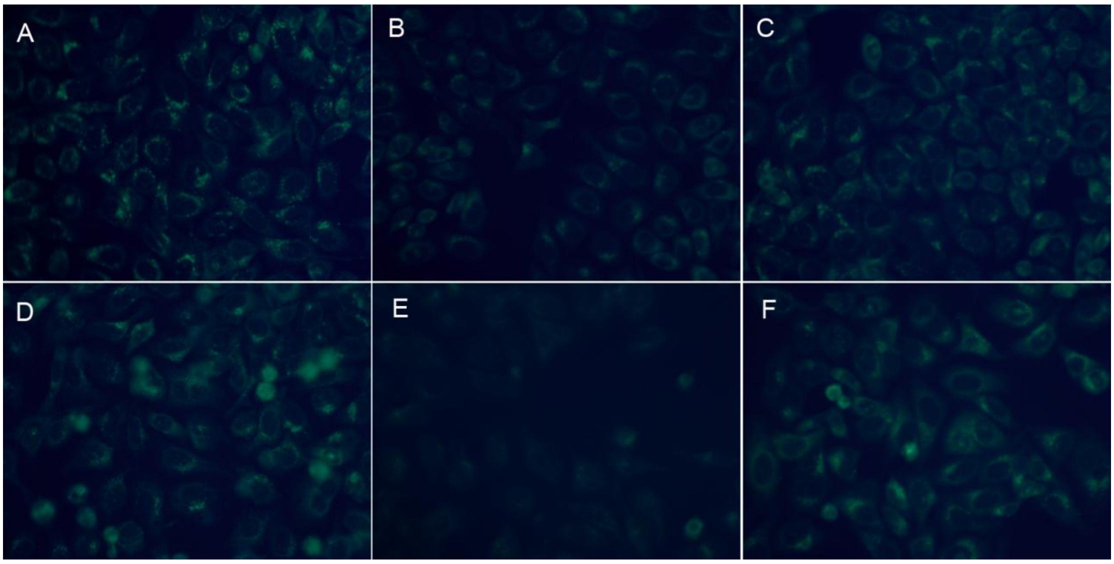

Fig. S4 The microcopic analysis of formation of autophagic vacuoles. (A) H_2O control; (B) 1.5 mM 3-MA; (C) 1.5 mM NAC; (D) 2 µM DpdtC; (E) 2 µM DpdtC + 1.5 mM 3-MA; (F) 2 µM DpdtC + 1.5 mM NAC.

DpdtC induced alteration in LMP

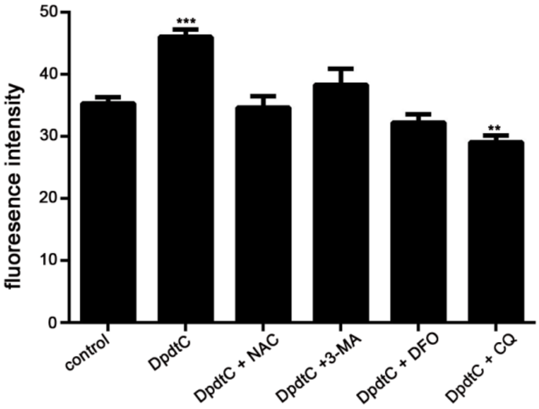

Fig. S5 The effect of DpdtC on lysosomal membrane permeability. The quantification analysis was performed by ImageJ. The results were obtained from three experiments. ***p< 0.01; **p < 0.05.
